# Analysis of Ultrasonic Vibration-Assisted Ball Burnishing Process on the Tribological Behavior of AISI 316L Cylindrical Specimens

**DOI:** 10.3390/ma16165595

**Published:** 2023-08-12

**Authors:** Eric Velázquez-Corral, Vincent Wagner, Ramón Jerez-Mesa, Jordi Lluma, J. Antonio Travieso-Rodriguez, Gilles Dessein

**Affiliations:** 1 Department of Mechanical Engineering, Universitat Politècnica de Catalunya, 08019 Barcelona, Spain; ramon.jerez@upc.edu (R.J.-M.); antonio.travieso@upc.edu (J.A.T.-R.); 2 Laboratoire Génie de Production, École Nationale d’Ingénieurs de Tarbes, 47 Avenue d’Azereix, 65000 Tarbes, France; vincent.wagner@enit.fr (V.W.); gilles.dessein@enit.fr (G.D.); 3 Department of Science and Materials Engineering, Universitat Politècnica de Catalunya, 08019 Barcelona, Spain; jordi.lluma@upc.edu

**Keywords:** vibration-assisted ball burnishing, tribology, residual stresses

## Abstract

In this study, we analyzed the effects of vibration assistance, combined with a ball burnishing process, in terms of topology, residual stresses, and tribological properties on 316L shafts. The burnishing variables consisted of the variation of the input force, the number of passes, and the activation of the vibration assistance, which is based on a 40 kHz frequency and 8 μm of vibration amplitude, derived in a screening design of three factors. The results show that the medium–high level of burnishing force, high level of the number of passes, and the activation of the vibration assistance are the best options in order to improve the average roughness, the microstructure, the increase in the compressive residual stresses, and the wear enhancement, besides all variables being significant in the *p*-value analysis through ANOVA. Statistically, the vibration-assisted ball burnishing improved the average roughness by 2.9%, enlarged the von Mises stress on the surface by 11.5% and enhanced the wear resistance of a 316L shaft and WC-Co ball contact up to 7.3%.

## 1. Introduction

Stainless steels are one of the most used materials for biomedical applications, such as prostheses or orthopedic implant manufacturing, through additive deposition [1,2] or traditional machining. However, for this purpose, most of the final workpieces need to enhance their mechanical properties, such as the wear resistance due to the friction present in the joints of a hip prosthesis [3] or lower corrosion by coating applications [4]. In particular, AISI 316L austenitic SS presents excellent corrosion resistance, high ductility, and good mechanical properties but poor resistance to wear, which often requires a surface upgrade. This enhancement is determined by surface integrity modification and improvement, so finishing operations have become a suitable solution for biomedical steel treatment.

Some surface deforming processes, such as burnishing or shot peening, have been widely used during the last few years to achieve the standard required by the biomedical community in terms of high wear resistance, durability improvement, and surface topology enhancement [5]. Mechanical surface treatments such as shot peening and burnishing are increasingly applied to biomedical parts to achieve the high requirements of surface finishing, surface hardness, wear resistance, and fatigue durability [6,7]. In particular, the use of the ball burnishing process in stainless steel parts for biomedical applications has grown significantly during the last few years [8,9,10]. This process consists of a cold deformation process, where the material is compressed using a rolling ball, obtaining a more regular and smoother surface and generating a hardened surface while keeping constant the dimensions of the part treated [11]. The ball burnishing can be divided into the elastoplastic flow of the material and the residual sink-in effect on each burnishing passage. Some of the authors reported a higher resistance to the wear between two sliding surfaces, a friction reduction, and a topology improvement by a material’s Gaussian redistribution [12,13,14]. Concerning the residual stresses, many authors declared the beneficial effects of increasing the compressive residuals in reducing crack propagation after applying a ball burnishing process [15,16,17]. In fact, there is a consensus that the increase in the burnishing force derives from a larger amount of residual compressive stresses at the surface, which benefits in slowing the crack propagation at the surface, enlarging its resistance to wear [18] or fatigue [19]. The ball burnishing process has also been described as an effective methodology to enhance wear resistance and reduce the coefficient of friction between two colliding surfaces [18]. More specifically, it was seen that the most important parameters to improve both properties were the burnishing force applied onto the surface and the number of passes performed, resulting in a hardness increase and a wear reduction. Attabi et al. [20,21] analyzed the wear enhancement and the surface integrity of 316L ball burnished specimens, finding a wear improvement of up to 65.2% compared to baseline material. It was also found that the increase in the number of passes had a big influence on surface hardness, which has a relation with friction reduction, while the improvement in resultant roughness also helped to reduce the coefficient of friction. However, it is also reported that despite all applied ball burnishing inputs helping to improve roughness, only the optimal ones reduced the friction compared to the baseline material.

One evolution of ball burnishing is vibration-assisted ball burnishing (VABB), which is based on the base physics of ball burnishing and the acoustoplasticity phenomenon studied by Blaha and Langenecker et al. [22]. The combination of both results in a larger material deformation due to the superimposition of the vibratory component on the deforming force applied onto the surface, deriving in a relaxation of the quasi-static stresses of the surface and, therefore, being easier to deform during the process. Teimouri et al. [23] studied the effects of the ultrasonic ball burnishing process on aluminum AA6061-T6 and concluded that the addition of the vibration assistance, using a 5 and 10 μm of frequency amplitude, improved the surface roughness by 15% and the superficial hardness by a 24%, respectively. In addition, it was found that the value of the compressive residual stresses increased deeper heights due to the ultrasonic impacts caused by the tool, and through this, the degree of mechanical working increased, and further refinement in microstructure was observed. This observation was also seen by Liu et al. [24], who described the greater influence of the microstructure, treated with VABB, as a consequence of the local severe deformation originated by the tool’s vibration when assisted.

Despite a lot of information available about the influence that ball burnishing has in terms of wear enhancement and the improvement of compressive residual stresses, there is a void space for vibration-assisted ball burnishing. Most VABB investigations are focused on surface integrity and residual stress enhancement, but not so many on wear enhancement or friction reduction. Generally, VA demonstrated positive applications to enhance the surface integrity factors within manufacturing processes, but its contribution in terms of tribo-characteristics is still controversial and not sufficiently investigated. For this reason, the main objective of this work is to analyze the impact analysis of the vibration assistance within a ball-burnishing process in terms of wear resistance enhancement and friction reduction on 316L ball-burnished stainless-steel shafts while confirming surface enhancement by this phenomenon. The results will be processed through an Analysis of Variance (ANOVA). This statistical tool permits quantifying the mean effects of the factors used on the response variables and evaluating the significance of each factor, then giving valuable information to determine the VA contribution to the final properties.

## 2. Materials and Methods

### 2.1. Specimens’ Preparation

The machining and finishing processes of the AISI 316L stainless steel used for this test (chemical composition shown in Table 1) are performed in a 3-axis CNC Machine Lathe “Pinacho SE 200 × 750” (Pinacho, Castejón del Puente, Spain).

The machining conditions prior to the burnishing were kept constant for all the specimens, being 60 m/min as cutting speed, 0.20 mm/rev as feed per turn, and a final diameter of 13 mm achieved in 3 passes. The insert used for this process was a rhombic 80° shape with a negative geometry CNMG120408-MM-YG213, made of carbide with a CVD TiCN+Al_2_O_3_+TiN coating, the clearance angle is set to 6°, and the rake angle to −6° once is assembled on the PCLNR 2020K 12 shank tool. The average quality roughness prior to burnishing was set to 1.5 ± 0.2 μm in order to obtain significant differences between all burnishing conditions applied to the specimens.

### 2.2. Ball Burnishing Equipment and Specimens

The vibration-assisted ball burnishing system used for this investigation is composed of the burnishing tool (the union of a base armor, a pre-load unit force based on a calibrated spring, one ultrasonic actuation unit built by a resonant ceramic piezoelectric excited to 40 kHz of frequency and an operational head formed by one 10 mm diameter 100Cr6 chromium steel ball at the top) and an external 40 kHz wave generator both plugged by a UFH connector, characterized by Fernández-Osete et al. [25]. The amplitude of vibration obtained at the top of the tool by the resonance of the system is about 8 μm.

**Figure 1:**
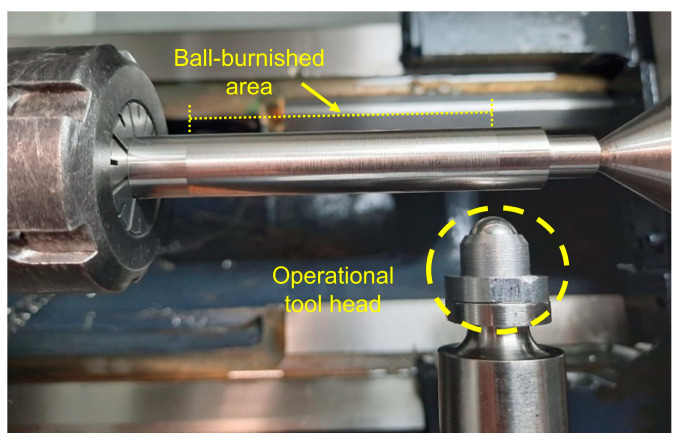
Burnishing tool head and specimen manufactured.

The definitive force applied onto the surface is a combination of multiple forces: the forces due to the irregularities of the material when the feed movement is performed, the force related to the vibration of the sonotrode when the assistance is applied, and the preadjusted force of the spring lodged. The final result of the process is shown in Figure 1.

### 2.3. Experimental Campaign and Specification

Based on the research group’s previous experience with the process on other materials such as AISI 1045 [12] or AISI301LN [26], a screening design of experiments was planned with the following factors and their corresponding levels:

- Burnishing force Fb: three numerical levels, from 80 N to 160 N with a central point in 120 N. Attabi et al. [21] found a surface degradation at slim plates with 240 N and a 10 mm ball, so by applying the radius corrections (in the function of ball mechanical and geometrical properties of the specimen) it is decided to apply a maximum force of 160 N to avoid surface degradation.
- Number of passes np: three numerical levels, from 1 to 5 passes with a central point in 3 passes.
- Frequency of the VA f: two different levels, being 0 or 40 kHz. Treated as a categorical variable.
- Burnishing speed: one level, being 2000 mm/min for all the specimens.
- Burnishing feed: one level, being 0.15 mm/rev for all the specimens.

The final experimental campaign resulted in a total of 12 specimens, and all combinations are shown in Table 2.

**Figure 2:**
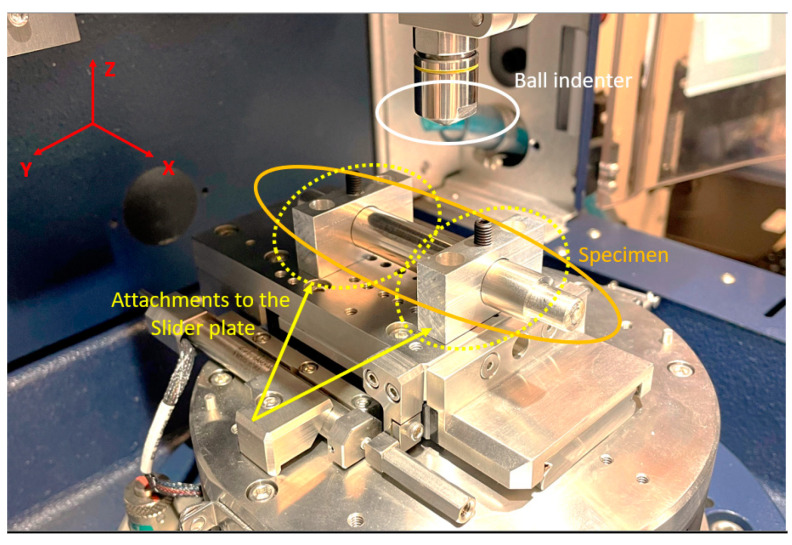
Tribology setup used for the testing.

The tribological procedure performed is based on Velázquez-Corral et al. previous work [12] and the ASTM G132-96 standard [27], see Figure 2. The conditions used are the following:

- Indenter characteristics: WC-Co 6 mm ball of diameter, with 600 GPa and 0.22 values for Young’s modulus and Poisson’s ratio, respectively.
- Specimen characteristics: AISI 316L SS 13 mm cylinder of diameter, with 200 GPa and 0.25 values for Young’s modulus and Poisson’s ratio, respectively.
- Physical inputs: Force applied of 20 N, making an imprint length of 20 mm, sliding at 10 cm/s during 5 min of testing.

During the execution of each test, the friction and input force is tracked and the *cof* (Coefficient of friction) is calculated in real-time at 100 Hz sampling frequency. The final acquisition is filtered using a low-pass filter, using a minimum-order filter with a stopband attenuation of 60 dB and compensating the delay introduced by the filter itself, with a passband frequency of 20 Hz. After that, the total worn imprint is measured using the 3D non-contact profilometer Alicona with a 10 nm of resolution, obtaining a 3D surface and measuring the volume difference between this surface and the one prior to the tribology testing, which defines the wear of the test in volumetric units.

### 2.4. Topology Acquisition

In order to acquire the surface and perform roughness measurements, it was used a non-contact 3D profilometer ALICONA Infinite Focus G5 (Alicona Imaging GmbH, Raaba, Austria) with an adaptive focus. The amplitude parameters measured prior to the tribology test are:

- Sa: Represents the arithmetical deviation of the roughness profile of the sampling length.
- Sq: Represents the root mean square of the heights.

### 2.5. Microstructure Analysis

Finally, Scanning Electron Microscopy (SEM) analysis was performed in order to see the microstructure evolution of the material for different burnishing, using an EVO HD15 by ZEISS (Zeiss and Quorum technologies, Oberkochen, Germany) equipped with an energy-dispersive X-ray spectrometer (SEM-EDX). Typical working parameters were an accelerating voltage of 15 kV and a working distance of 10 mm.

### 2.6. Residual Stress

In order to evaluate the residual stresses onto the treated surfaces, it is used an X-ray diffraction method based on applying Bragg’s law while quantifying the change in the inter-planar spacings. The experimental application was performed with a V-α anode, 600 s of exposition time, and 20 measures. The post-processing method was curve fitting vs. sin²(ψ).

**Figure 3:**
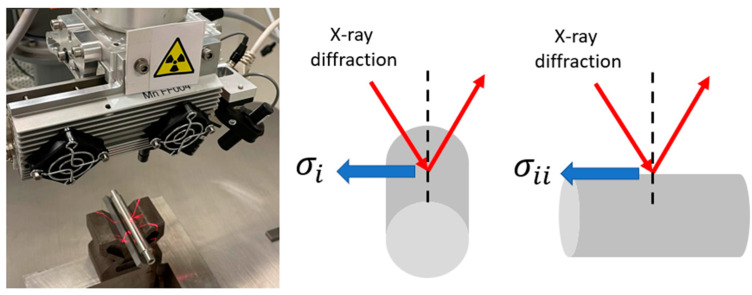
Residual stresses measurement unit and setup for cylindrical samples.

The application of the test was delivered in terms of the residual stress tensor. In which *σ_i_* corresponds to the tangential direction and *σ_ii_* corresponds to the axial direction of the specimen’s generatrix (see Figure 3), *τ*_12_ and *τ*_21_ are the shear stresses.

(1) $$\sigma =\left[\begin{array}{cc} \sigma_{i} & \tau_{12}\\ \tau_{21} & \sigma_{ii} \end{array}\right]$$

However, another indicator may be included to analyze the general residual stress state of the surface, the von Mises stress which is described in Equation (2).

(2) $$\sigma_{vm}=\sqrt{\sigma_{i}^{2}+\sigma_{ii}^{2}-\sigma_{i}\sigma_{ii}+3\tau_{12}\tau_{21}}$$

## 3. Results

### 3.1. Topography

**Figure 4:**
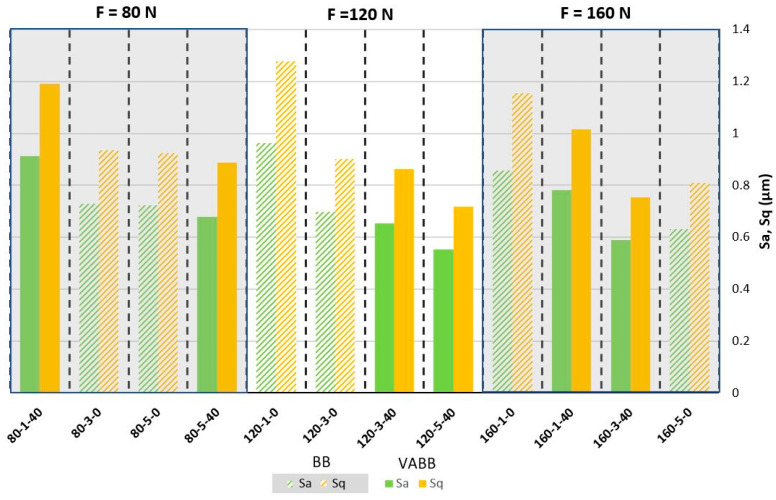
Three-dimensional Roughness results for 316L specimens.

The Sa and height parameters were measured according to the ISO 25178 Standard [28]. The results are presented in Figure 4.

Results show a reduction of the Sa and Sq when the number of passes is increased, and the vibration assistance is activated. It is clear that there is a decreasing tendency of the average and maximum roughness from lower levels of the number of passes plus the non-addition of vibration assistance to high levels of the number of passes and the vibration assistance activated [29]. It seems that the number of passes has the more significant importance in order to enhance the surface in terms of roughness, having a great association with the vibration assistance. When looking more carefully, it can also be appreciated that when those medium–high levels of the number of passes are applied, the input force seems to enhance the topology when this one is increased. For example, comparing the specimens 80-5-40 vs. 120-5-40 or 120-3-40 vs. 160-3-40, it is noticed a roughness improvement by only increasing the input force. The best combinations obtained are 120-5-40, 160-3-40, and 160-5-0 (obtaining 63%, 61%, and 58% of Sa enhancement, respectively, as it is shown in Table 3), in that order, which may indicate that the number of passes is the most significant parameter and, combined with the vibration assistance and a high force, boosts the final surface roughness improvement.

As can be seen, roughness is reduced by increasing the degree of plastic deformation by means of the augmentation of the three burnishing inputs analyzed. In particular, the increase in the burnishing force seems to not exceed the material limit at high forces, meaning that there could still be scope for force increase to obtain optimized roughness. The roughness trend is descending if an equal number of passes and VA value pairs are compared, as is seen by analyzing 120-3-0 with 80-3-0 or 160-3-40 with 120-3-40, so it is confirmed the positive effect of increasing the burnishing force to enhance the resultant roughness. In similar studies, Attabi et al. [21] obtained average roughness improvement with 240 N of force with a 10 mm ball but also experienced texture deterioration (compared to this optimal value) if the ball diameter was reduced, thus increasing the equivalent contact pressure. The descending trend of roughness when the number of passes and the VA is applied is evident and agrees with the bibliography [30,31]. In terms of force and number of passes increase, improvement can be explained by the fact that the ball is rolling and smoothing out the bulged edges of the initial machining or the previous pass, so the probability of deforming the asperities is increased by augmenting the force and performing successive burnishing passes; thus, the surface is being smoothed at each pass. Concerning the VA, the kinematic energy added to the process enhances the work-hardening but to a lower degree than the burnishing force [32], explaining why VABB roughness values are better than BB when the same level of plastic deformation (burnishing force and several passes) is applied.

**Figure 5:**
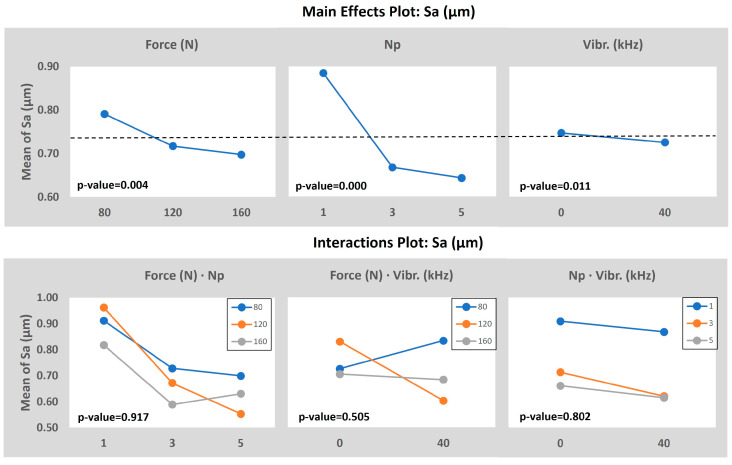
ANOVA results taking the Sa as the output value.

The mean effects and the interaction plots of the model, taking the Sa as the output, are presented in Figure 5. First of all, the *p*-value analysis determines that the number of passes, the burnishing force, and the vibration assistance, in that order, are the most important and significant parameters due to having a *p*-value lower than 5%. However, none of the interactions analyzed are significant enough to be considered, so they are rejected as a hypothesis. The 0.000 *p*-value registered for the number of passes means the huge impact that this variable has on the final value of the average roughness.

As it was alleged in the experimental results section, the increase in the force and the number of passes, plus the addition of the vibration assistance, improve the final topology of the specimen after the ball burnishing process within the parameter interval chosen in this study.

### 3.2. Microstructure Analysis

**Figure 6:**
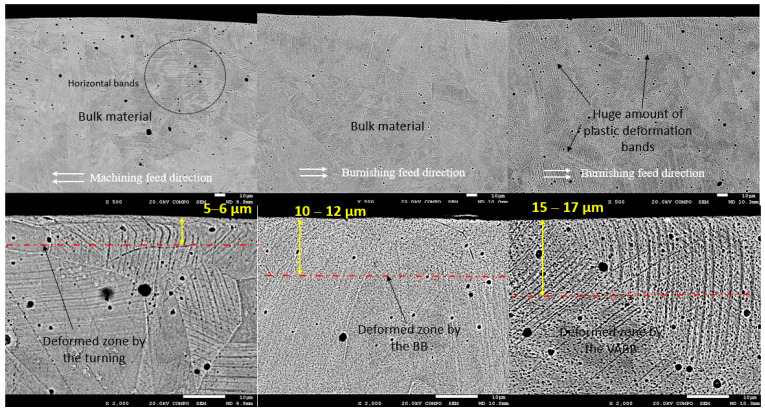
Cross-section microstructures by columns: 1st corresponds to machined surface, 2nd column corresponds to the 80-5-0 sample (BB), and 3rd column corresponds to the 80-5-40 sample (VABB).

The microstructures shown in Figure 6 correspond to a cross-sectional part of samples 80-5-0 for ball burnishing (BB) and 80-5-40 for vibration-assisted ball burnishing (VABB). It also extracted a non-burnished sample section in order to make a comparison between the three resultant microstructures. The general images (see Figure 6, 1st column) show a perspective of austenite grains, which defines the general microstructure of these AISI 316L specimens.

Firstly, the M specimen reveals some plastic deformation boundaries at the coating due to the shearing induced during the turning. It is visible that the grain orientation of these remarked boundaries (see Figure 6 first column) is quite more horizontal compared to other specimens at 30 μm of depth, showing no excessive deformation compared to other processes. More in detail, when the coating is zoomed, it can be revealed that there is a change in the plastic deformation boundaries in the same direction as the turning feed, estimated on 5–6 μm of depth and then defining the affectation profundity. Regarding the BB specimen, it can be seen a major density of plastic deformation traces, or plane slides, in higher depths compared to the M specimen. According to the bibliography [33], these bands present in all 316L surfaces are caused by different degrees of plastic deformations on the austenite grains. These deformations are compatible with a twinning formation microstructure on a nanometric scale [33], which could induce nanograin refinement and, combined with a dislocation network similar to the present on these specimens, could derive hardness enhancement and the increase in the compressive residual stresses due to the exerted pressure on the surface. Looking in detail at the top coating, it is visible that the deformation caused by the ball is pushing the material toward the center of the specimen, thus homogenizing the surface compactness and achieving a depth of affectation around 10–12 μm. Finally, the VABB specimen shows a huge density of plastic deformation traces along the entire surface. This mechanism of deformation is enhanced by the application of the VA, which combines the acoustoplastic phenomena plus the superimposition of dynamic and static forces during the process, thus exerting more pressure and deforming the material. In this case, the coating deformation due to the VABB process is estimated at 15 μm, but the higher presence of parallel bands at deeper depths points to a higher effect.

### 3.3. Residual Stress

**Figure 7:**
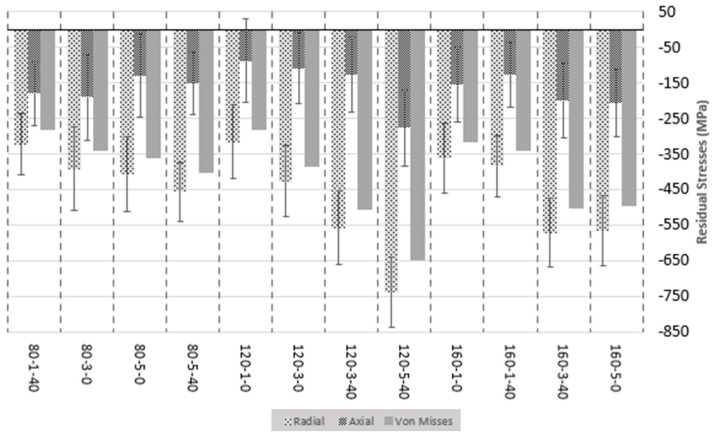
Residual stress results for all the combinations.

The residual stress measurement was made to obtain both stress components, *σ_i_* and *σ_ii_*, and Equation (2) was applied in order to calculate von Mises’s stress value. The results obtained are presented in Figure 7.

First of all, results show that compressive (negative) stress values are present in all specimens, which may prevent crack growth in fatigue regimes, confirming that positive property. In addition, no positive value of stress is reported, which is caused by the shearing suffered during the previous machining process. Therefore, the burnishing totally counteracts the effect of the machining in terms of stress, having a value near the 550 MPa of von Mises stress.

Secondly, it is also reported that the tangential direction of stress measuring, *σ_i_* is higher than the axial direction of stress, *σ_ii_*. This can be explained due to the fact that the burnishing input force applied in a normal direction onto the surface (X-axis in a lathe) is way higher than the dragging force applied due to the feed of the tool (Z-axis in a lathe) during the burnishing operation and, therefore, causing more deformation that relates directly to the stress.

The increase in the residual stresses is directly associated with the amount of strain induced during the plastic deformation. Several investigations pointed out that the pressure exerted on the surface was the most important parameter to induce compressive residual stresses [34,35] by means of augmenting the amount of strain and the depth of affectation. Indeed, this plastic deformation is previously described as a combined effect of static force, the number of passes exerted, and the superimposition of the dynamic component of the force (VA). The process consists of inducing this plastic deformation (after surpassing the initial elastic deformation) through the rolling ball pressure onto the surface, axial and tangential to the generatrix of the shaft. The material flow and redistribution caused by shearing provoke an increment in the stress-strain field in the surface and subsurfaces. Next, the work hardening produced is caused by the dislocation movement and rearrangement of the inner crystals, known as dislocation density evolution, and concludes at grain refinement by means of dynamic recrystallization. When the rolling ball is no longer pressurizing, the remains of the stress are relaxed and converge to residual stress. In terms of the number of passes, some investigations declared that the parameter is not significant enough [17]. However, as was seen with roughness results previously presented, burnishing force values used seem to be quite low to heavily deform the AISI 316L texture. Residual stresses are directly dependent on the plastic deformation degree and work hardening and increase when a higher number of passes is applied [36]. Regarding the VA, Teimouri et al. [37] state that VA enhances the plastic flow stress by means of dislocation drag and thermal activation, concluding that increasing the vibration amplitude results in further strain and strain rate because of a greater value of deformation radius (augmented by the increase in the kinetic energy) and impact velocity. Furthermore, the author points out that the increased ultrasonic energy density at further vibration amplitude contributes to the reduction of activation energy and subsequent flow stress. The recrystallization is also enhanced by the softening effect during the plastic deformation, thus enhancing the strain induced [37]. This affirmation correlates with the results obtained in terms of microstructure, where the VABB specimen showed a higher amount and greater effect of deformation mechanisms than the BB specimen. Therefore, the increase in plastic deformation caused by the VA is greater and favorable for the mechanical properties and surface integrity factors [23]. In general, residual stresses are defined as a complex distribution of mechanical, thermal, and metallurgical effects simultaneously that often actuate synergistically.

**Figure 8:**
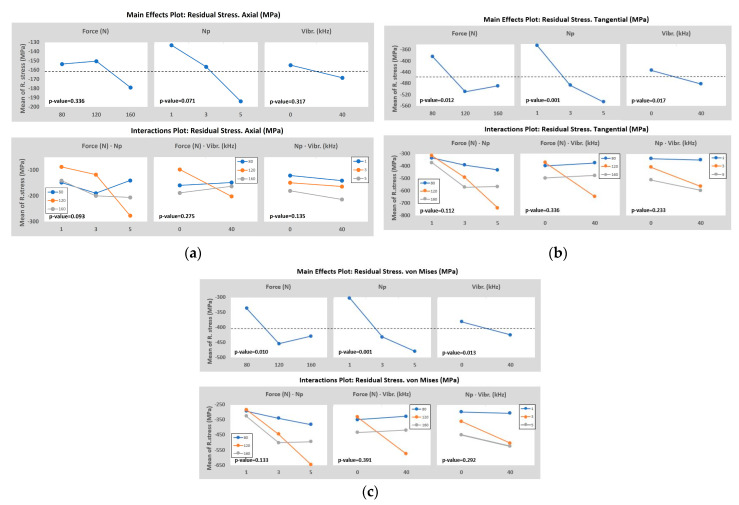
ANOVA results taking the: (**a**) Axial; (**b**) Tangential; (**c**) von Mises; residual stress as the output value.

When analyzing the tendency of the von Mises calculated values, it is quite clear that the effect enhances the induction of higher negative values when the combination of a high number of passes and the activation of vibration assistance is applied. The force, in this case, seems to be less significant than the other input parameters, but it has the desired effect as it increases. The best combinations are 120-5-40, 160-3-40, and 120-3-40, demonstrating that also the VABB enhances more than BB. The mean effects and the interaction plots of the model, taking the Residual stresses of both components measured and the von Mises stress as the output, are presented in Figure 8.

The results of the tangential direction (see Figure 8b), corresponding to *σ_i_*, determine that the number of passes, burnishing force, and the addition of the vibration assistance, in that order, are significant. When the number of passes increases, the value of the residual stress increases, and the same is applied to the vibration assistance. In the case of the force, it is appreciated that the best point is 120 N instead of 160 N. This happens due to the best combination reported is 120-5-40 and is especially higher than the rest, so the mean of the 120 N force group is higher than 160 N. However, looking at the evolution of both groups, if the design of experiments considered higher levels of force (160 N or more) with a high number of passes and vibration assistance, the expected result may be higher than the 120-5-40. The results of the Axial direction (see Figure 8a), corresponding to *σ_ii_*, determine that the number of passes, the interaction of the burnishing force plus and the number of passes, and the interaction of the burnishing force plus and the number of passes, in that order, are the most important parameters but being not significant enough. All *p*-values reported are higher than 0.05. This concludes that no parameter used has a special effect on the second direction and other input parameters. This could be explained by the fact that burnishing deformation is mainly performed in the normal direction to the surface, while the forces in the perpendicular direction to the surface are lower and then provokes less deformation. This effect was also observed by Chomienne et al. [17], who observed that plastic deformation is not that intensive in both directions during a single revolution. In the circumferential direction, the work material is always deformed in the same direction, whereas in the axial direction, the work material is deformed in two different directions. Von Mises stress values (see Figure 8c) are almost completely influenced by the first component of the stress *σ_i_*, so both plots’ graph results are essentially the same, changing a bit the *p*-values rates but not the grade of significance.

In terms of residual stresses, it is reported an increase in the von Mises residual stress at the surface by increasing the burnishing force and the number of passes, also applying VABB instead of BB to enhance them, which is estimated in an 11.5% increase. These results are within the scope because increasing the burnishing force causes a larger plastic deformation and is boosted by the vibration amplitude of the assistance. The number of passes has been reported as another very influential parameter to enhance the residual stresses within a burnishing process [38]. In conclusion, the combination 120-5-40 is the best to maximize the compressive residual stresses, either tangential or axial to the specimen, thus the equivalent von Mises stress.

### 3.4. Frictional Performance

**Figure 9:**
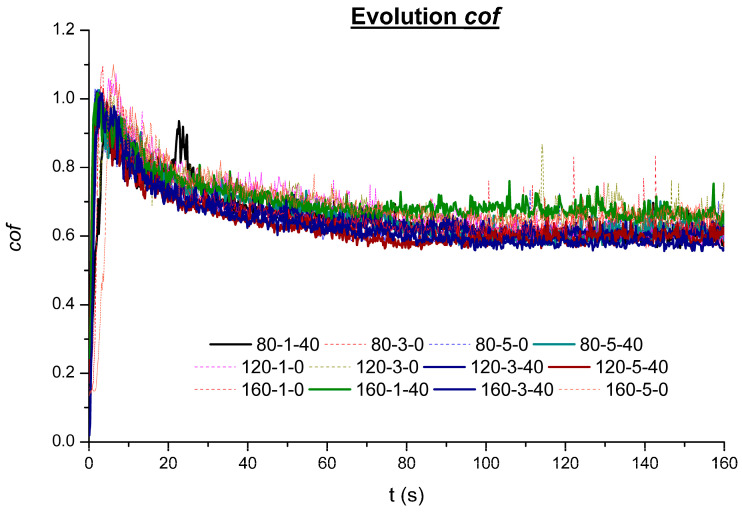
Evolution of the *cof*.

The tests performed under dry conditions in the tribometer concluded that all tests have a coefficient of friction evolution between 0.6 and 1.1 (see Figure 9). The *cof* evolution corresponds to the evolution along all the tests and the stationary period. All tests executed show a fast increase during the first seconds of the test, even arriving to surpass the *cof* = 1.0, and then start decreasing while the initial track is formed and the formation of the debris from the surface asperities of each specimen.

**Figure 10:**
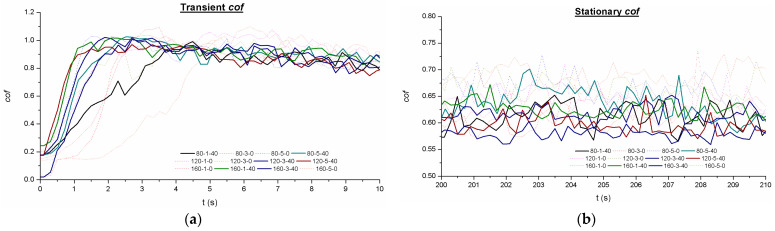
*cof* plot for the: (**a**) Transient; (**b**) Stationary.

Therefore, as can be seen in Figure 9, the first 40–60 s of execution is part of the transitory state, and then the stationary state initiates. When analyzing the results, it can be seen that VABB specimens (continuous lines) are evolving homogeneously during the first stages and, especially after 4–5 s, show similar results. In fact, after 5 s of testing, all specimens enter the first stage of stabilization within the transient mode; they will start to decrease the friction force along the time but in the same trend until the 40–60 s of the steady-state period. Figure 10a shows that the maximum peak of friction corresponds to the BB specimen (dotted lines); when this first stabilization point is reached, BB remains systematically over the VABB, pointing to a friction reduction when using VA. This apparent improvement caused by friction reduction could be related to the higher degree of deformation of the AISI 316L microstructure for VABB specimens, where it was observed a major presence of deformation bands at major depths, which is caused by a bigger accumulation of deformation energy that could be a symptom of a dynamic recrystallization activation. Therefore, grain refinement derives a hardening effect and higher residual stresses. As is seen in previous sections, VA increases the values of compressive residual stress, so it makes sense that friction reduction is enhanced due to the VA application. Following the previous explanation, it seems that VA may enhance friction reduction during the steady-state period, and this trend is kept during the posterior decrease in the *cof*. Figure 10b shows that the average stationary coefficient of friction is between 0.55 and 0.72 for all specimens, and again it is visible that VABB is systematically less frictional than BB specimens. These results agree with other bibliographic references where it is tested the reduction of the friction force when VA is applied [39,40]. The comparison between similar pairs, such as 120-3-40 and 120-3-0 or 80-5-40 and 80-5-0, shows that the average *cof* is reduced when the specimens are VABB, so it could be said that VABB enhances friction reduction but under a limited range and within the tribo-setup conditions tested. Again, no particular relation between roughness parameters and *cof* is found, so it seems that the main contributors to the friction reduction are residual stresses enhancement and microstructure evolution for VABB specimens. VA seems beneficial to reduce the friction force during the fretting test, which agrees with the bibliography [39,40].

### 3.5. Wear Resistance Analysis

**Figure 11:**
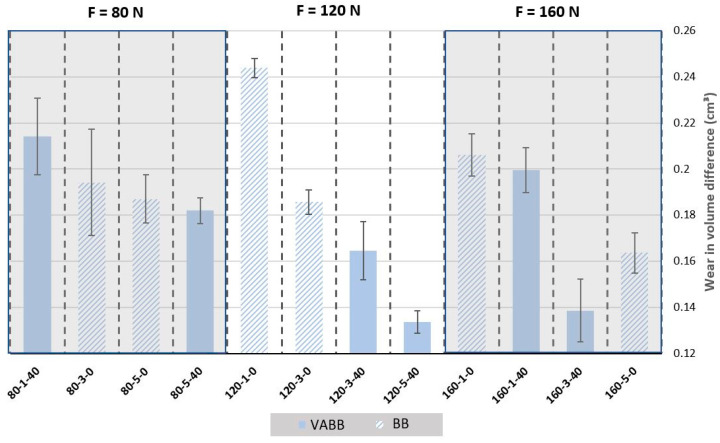
Wear results for 316L specimens.

The results obtained after the tribology and surface acquisition tests are presented in Figure 11.

The plot shows that, at first glance, the number of passes and the activation of the vibration assistance have a positive effect in terms of reducing the wear after the tribology tests. However, the burnishing force may improve the results but not for all combinations. For example, the worst specimen in terms of wear resistance is 120-1-0, but the best one is 120-5-40, making the most accentuated evolution of the different levels of forces applied. However, despite this first sample, if equal samples in terms of force are compared, such as 80-3-0 vs. 120-3-0 or 120-3-40 vs. 160-3-40, it is observed that increasing the burnishing force helps enhance the wear resistance. The same happens with the addition of vibration assistance, with 120-3-0 vs. 120-3-40 as a good example of it. The use of a low number of passes is clearly not improving as much as the other combinations. Despite experimenting with a lower standard deviation than other parameters, it is still difficult to determine if the number of passes and the addition of the vibration assistance enhances the wear resistance always and if they are significant, so for that, an ANOVA will be performed.

**Figure 12:**
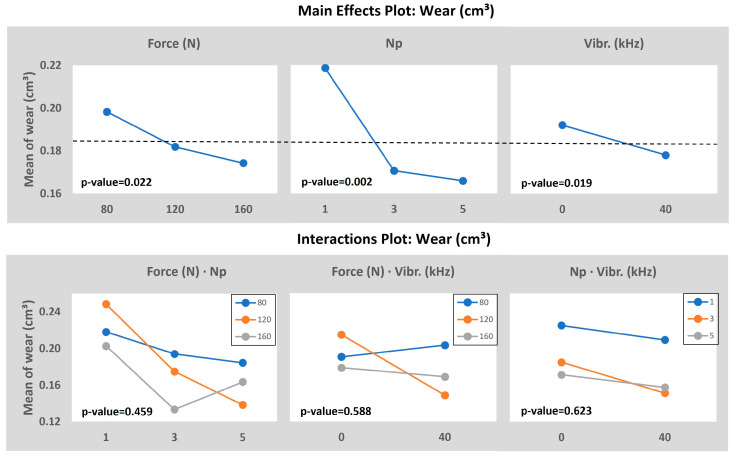
ANOVA results taking the Wear as the output value.

The mean effects and the interaction plots of the model, taking the final Wear as the output, are presented in Figure 12. First of all, the *p*-value analysis determines that the number of passes, the vibration assistance, and the vibration burnishing force, in that order, are the most important and significant parameters due to having a *p*-value lower than 5%. However, none of the interactions analyzed are significant enough to be considered. The 0.002 *p*-value registered for the number of passes means the huge impact that this variable has on the final value of the average roughness.

This investigation points to a wear enhancement by adding VA to a conventional ball burnishing process on 316L stainless steel shafts, which is estimated at a 7.3% of statistical improvement within the input range selected. It is found that a medium–high number of passes, the addition of the VA, and a high burnishing force is the best combination possible of the tested. The residual stress-wear relation found for this set of parameters hints that when residual stress is boosted, which is mainly caused by an increment of the plastic deformation at the surface due to the burnishing, the wear resistance is enhanced too. In fact, it was known that the increase in the burnishing force derives from a hardness increase, with a more homogeneous and compacted microstructure, which is the main consequence of a wear enhancement between two colliding surfaces [18]. Furthermore, the number of passes also increases the superficial hardness by plastically deforming several times in the same spot, obtaining a more condensed grain microstructure. The VA is also described as a hardness increase factor in terms of depth of affectation and helping the incrementation of plastic deformation, thus boosting the effectiveness of the burnishing force-number of passes set. In addition, the reduction of the superficial roughness leads to a wear enhancement of 316L shafts [21], then makes sense that the VA really enhances the wear.

In terms of tribological properties enhancement, it can be interpreted that *cof* and wear are enhanced when VA is applied, mainly by a major impact of the compressive residual stress induction. Furthermore, the deformation features revealed in the microstructure pointed out major compactness, homogeneity, and depth of affectation by means of the VA. The possible formation of nano-structures in the form of twinnings and dynamic recrystallization can contribute to wear resistance by accommodating deformation and preventing crack initiation and propagation. Plastic deformation helps distribute the applied load and reduces the concentration of stress at localized points, which can reduce wear and may explain the evolution of both properties when the burnishing inputs are augmented. The work-hardening induced strengthens the material subsurfaces by increasing dislocation density and promoting grain refinement, making the surface more resistant to wear.

## 4. Discussion

The roughness results determine that the number of passes is the most influential parameter and is followed by the burnishing force, which coincides with some bibliographic references with the same material [21]. It has also been proven that VABB offers better results than BB in terms of surface quality, as was also expected [41,42].

Micro-structural results present deformation due to the shearing of machining and BB/VABB processes. It is reported an increase in the depth of affectation when the VA is applied, and, especially, a major presence of deformation mechanisms expressed as plan sliding. These deformations are compatible with a twinning formation microstructure on a nanometric scale [33], which could induce nanograin refinement and, combined with a dislocation network similar to the present on these specimens. This assumption makes sense after noticing an improvement in the compressive residual stresses and roughness when applying VA. However, further confirmations should be made in the future by performing EBSD and TEM analysis at the surface to understand better the overall plastic deformation.

In terms of residual stresses, it is reported an increase in the von Mises residual stress at the surface by increasing the burnishing force and the number of passes, also applying VABB instead of BB to enhance them, which is estimated in an 11.5% increase. These results are within the scope because increasing the burnishing force causes a larger plastic deformation and is boosted by the vibration amplitude of the assistance [30]. The number of passes has been reported as another very influential parameter to enhance the residual stresses within a burnishing process [38].

The results in terms of *cof* show no heavy variation between specimens, which may indicate that the input range of values is not very influential. However, results displayed that may exist a friction reduction when VA is applied, as it is shown in Figure 10, during the stationary zone. This could be explained due to the fact that it is demonstrated that friction reduction is directly related to the initial roughness by decreasing as well as roughness [30]. Therefore, by modifying the burnishing force and number of passes to wider values, maybe a more incisive conclusion can be achieved in order to see if the VA really boosts the performance or if it is insignificant in terms of friction reduction. Furthermore, it was kept constant the counter-part material and the tribology testing force, so more tests should be performed in this field to arrive at a definitive conclusion about the VA within this VABB-tribology combination.

The main contribution of this investigation points to a wear enhancement by adding VA to a conventional ball burnishing process on 316L stainless steel shafts, which is estimated at a 7.3% of improvement within the input range selected. It is found that a medium–high number of passes, the addition of the VA, and a high burnishing force is the best-performing combination of all tested. The residual stress-wear relation found for this set of parameters hints that when residual stress is boosted, which is mainly caused by a hardness increase in the surface due to the burnishing, the wear is enhanced too. In fact, it was known that the increase in the burnishing force derives from a hardness increase, with a more homogeneous and compacted microstructure, which is the main consequence of a wear enhancement between two colliding surfaces [18]. Furthermore, the number of passes also increases the superficial hardness by plastically deforming several times in the same spot, obtaining a more condensed grain microstructure. The VA is also described as a hardness increase factor in terms of depth of affectation and helping the incrementation of plastic deformation, thus boosting the effectiveness of the burnishing force-number of passes set. In addition, the reduction of the superficial roughness leads to a wear enhancement [21] of 316L shafts, then makes sense that the VA really enhances the wear. Indeed, the VABB microstructure pictures revealed a higher density of plastic deformation plans or traces. This is caused by a more intense stress field due to the compression exerted by the ball during the process, and the depth of affectation is enhanced by the easiness of dislocation inductions during the assistance. This compressive field, observed as an increase in the compressive residual stress, actuates as a protector coating by means of a potential hardness increase on the surface, thus reducing the implicit wear and sliding force during the tribology testing.

## 5. Conclusions

With all the tests and analyses performed, it can be concluded that

- Ball burnishing improves the average roughness of 316L shaft surfaces, and the vibration assistance improves this property, statistically, by 9% in average terms. The best parameters found in this study correspond to 160 N of force, five passes, and the activation of the vibration assistance.
- The coefficient of friction seems to be similar for all specimens tested. However, it is noticed that the vibration assistance could reduce up to 2.2% despite not being statistically significant.
- The increase in the residual stresses in the tangential direction is directly dependent on the number of passes and the force applied, while the axial seems to be similar for all specimens. The VA enhances the von Mises stress value by 11.5% compared to conventional ball burnishing.
- The wear decreased by 7.3% when using VABB instead of BB. It is also reported that the increase in the number of passes and the burnishing force helps to enhance its resistance. It is also found that there exists a relation between the von Mises residual stress and the wear of the tribology testing.

These results confirm the positive effect of vibration-assisted ball burnishing compared to conventional ball burnishing in terms of wear resistance and increase in residual stresses in 316L shafts. However, further questions can be made starting with the analysis of the microstructure evolution and depth affectation when using different burnishing parameters. This may help to understand the hardness profile in depth and add more complementary information to the residual stresses and the wear reported, which may be related. Another point of focus is to perform a wider range of burnishing parameters and see if there is a variation in the coefficient of friction once the microstructure is analyzed by performing EBSD and TEM tests.

## Figures and Tables

**Table 1:** Nominal chemical composition of 316L SS.

Material	Chemical Composition by Element (wt. in %)									
AISI 316L	Fe	C	Si	Mn	P	S	Cr	Ni	N	Mo
	Bal.	0.03	1.00	2.00	0.05	0.02	17.50	11.50	0.10	2.00

**Table 2:** Design of Experiments for the burnishing operation.

Nomenc.	*Fb* (N)	*np*	*f* (kHz)
80-1-40	80	1	40
80-3-0	80	3	0
80-5-0	80	5	0
80-5-40	80	5	40
120-1-0	120	1	0
120-3-0	120	3	0
120-3-40	120	3	40
120-5-40	120	5	40
160-1-0	160	1	0
160-1-40	160	1	40
160-3-40	160	3	40
160-5-0	160	5	0

**Table 3:** Sa improvement for each specimen compared to the initial surface after machining.

Nomenc.	Sa (μm)	ΔSa (%)
80-1-40	0.912	39
80-3-0	0.729	51
80-5-0	0.723	52
80-5-40	0.678	55
120-1-0	0.963	36
120-3-0	0.699	53
120-3-40	0.654	56
120-5-40	0.554	63
160-1-0	0.856	43
160-1-40	0.781	48
160-3-40	0.590	61
160-5-0	0.631	58

## Data Availability

The raw/processed data required to reproduce these findings cannot be shared at this time, as the data also form part of an ongoing study.
